# Supplementary material for: Reconciling patterns of long-term topographic growth with coseismic uplift by synchronous duplex thrusting
Source: Nat Commun. 2023 Dec 6;14:8073. doi: 10.1038/s41467-023-43994-6 (PMC10700525; doi:10.1038/s41467-023-43994-6)
Supplement: Supplementary file 1 — Supplementary Information [file 41467_2023_43994_MOESM1_ESM.pdf]

Supplementary information

for

Reconciling patterns of long-term topographic growth with coseismic uplift by synchronous duplex thrusting

Yuqing Zhang[1,2,3], Hanlin Chen[2,3]*, Xuhua Shi[2,3,4]*, Rafael Almeida[5], Richard Walker[6], Xiubin Lin[2,3], Xiaogan Cheng[2,3], Hongdan Deng[2,3], Zhuxin Chen[1], Xiu Hu[7]

[1]Research Institute of Petroleum Exploration and Development, PetroChina, Beijing, China
[2]Key Laboratory of Geoscience Big Data and Deep Resource of Zhejiang Province, School of Earth Sciences, Zhejiang University, Hangzhou, China
[3]Research Center for Structures in Oil and Gas Bearing Basins, Ministry of Education, Hangzhou, China
[4]Xinjiang Pamir Intracontinental Subduction National Observation and Research Station, Beijing, China
[5]Department of Geological Sciences, San Diego State University, San Diego, USA
[6]Department of Earth Sciences, University of Oxford, Oxford, UK
[7]Guangdong Provincial Key Laboratory of Geodynamics and Geohazards, School of Earth Sciences and Engineering, Sun Yat-Sen University, Zhuhai, China

*Authors for correspondence, e-mail: hlchen@zju.edu.cn; shixuhua@zju.edu.cn

Contents of this file

Text S1 to S3
Tables S1-S2
Figures S1-S9

Text S1. More description on methods

S1.1 Modeling of growth strata

The growth strata model is constructed at the front of a duplex system, which is characterized by the upward motion of the root thrust and the downward motion of the roof thrust during the synchronous thrusting of two ramp faults. Therefore, this type of growth strata records both thrusting and normal-faulting-like processes, and provide an excellentmarker for synchronous duplex thrusting. In our modeling, we use both sharp and smooth fault-bends in the duplex front for better comparison with published growth strata models and case field examples, respectively.

We use the 2D MOVE^{TM} software (PE Limited), available at https://www.petex.com, to generate the growth strata in six steps, each with equal fault slip and syn-sedimentary thickness. Moreover, the roof thrust and root thrust are featured by constant fault slip in each step. These constant parameters simplify the model and enable us to focus on the general feature of growth strata during synchronous activity of two adjacent faults in the duplex system.

During the modeling, true synchronous thrusting on two faults not possible because MOVE software only allows one fault to move at atime. Thus, synchronous motion on the two faults is modeled as a quasi-continuous process by incremental finite slip on each fault (Fig. S1). Kinematically, the frontal ramp activity was modeled with the fault-bend fold (FBF) technique, while the rear fault was modeled under the fault parallel flow technique due to this fault containing a segment of downward slip (in the upper ramp) that cannot be modeled by the FBF technique in the MOVE software. In addition, we construct the reference model exclusively controlled by the frontal ramp fault with all other parameters equal to the synchronous thrusting model. The detailed parameters of the growth strata modeling are presented in Table S1. All original files obtained through modeling in the MOVE software are provided in the Source Data.

S1.2 Modeling of terrace folds

The terrace fold models have been constructed in a duplex system that is comprised of two detachment layers and two ramp faults in between (Fig. 2c-2f). In this duplex system, flat faults (detachments) and ramp faults were connected by smooth fault-bend in order to simulate the terrace folds in the field. We insert five flights of terraces (T0 to T4) above the duplex system during the incremental deformation. Among these terraces, T0 is the youngest and yet to be deformed, while T4 is the oldest that contains the highest terrace fold.

In our models, we use 2D MOVE software to produce the terrace folds (T1 to T4) in four steps, each with an equal fault slip of 60 m. This amount of slipis arbitrary, but the model results show similar patterns regardless of the amount of slip. Models 1 to 3 share the same first three steps but differ in the last step. In the first three steps, the amount of fault slip is equally separated into the rear ramp (30 m) and frontal ramp (30 m) to simulate the synchronous thrusting of the duplex system. In the last step, Model 1 remains the same as the former steps; Model 2 is featured by the cease of slip on the rear ramp (F2), while the frontal ramp remains active and accommodates all the fault slip; Model 3 is featured by the cease of slip on the frontal ramp (F1), while the rear ramp remains active and accommodates all the fault slip.

Similar to the growth strata model described above, synchronous motion on the two faults is modeled as a quasi-continuous process by incremental finite slip on each fault; see Fig. S5 for the details. Kinematically, the frontal ramp activity was modeled by the fault-bend fold (FBF) technique, which is widely used in the kinematic modeling of thrust systems with flat-ramp-flat geometry. However, the rear ramp was modeled with the fault parallel flow technique because this fault contains a downward slipping segment (in the upper ramp) that cannot be modeled by the FBF technique in the MOVE software. Nevertheless, the kinematic difference between the rear ramp and frontal ramp in an individual model does not influence our results because we focus on geometric variations between the three models. In the reference model, only the frontal fault is active. Thus, this fault accommodated all the fault slip in the four steps and

controlled the deformation of the terrace folds.

The detailed parameters of the terrace folds modeling have been presented in Table S2. All original files obtained by modeling in the MOVE software are provided in the Source Data.

Text S2. Sesimic reflection data and geologic profile

The seismic reflection profile presented in this study was acquired by the Tarim Oilfield Company (PetroChina) and processed in 2004. The seismic data were recorded to 7-8 s two-way travel time at 2 ms sampling rate. The vertical seismic resolution is about 10-25 m for Cenozoic sequence and about 16-38 m for Paleozoic-Proterozoic sequences. We converted the pre-stack time migrated sections into depth sections using seismic velocities (Table S3) derived from well data[1]. We then combine the depth-converted seismic reflection profile with the surface geology to construct the geological cross section by using fault-related folding technique (Fig. 1d in the manuscript).

Text S3. Modeling of terrace folds above the front ramp in the Pishan belt

We construct models of terrace folds above the frontal ramp to determine whether the penultimate ramp fault (F5) remains active during the formation of the lowest terrace fold (Tw1). The terrace folds were produced using the FBF process in 2D MOVE software. Terrace models folded with 0 m, 50m, 80 m, 90 m, 100 m, 110 m, 120 m, 150 m, and 200 m of fault slip are shown. By comparing the modeling results with the field observation from Ainscoe et al.[2], we find that these two are most consistent when the amount of fault slip is around 100 m (Fig. S9). This consistency suggests that the penultimate ramp fault ceased activity when the terrace Tw1 began to deform (Fig. 4a in the main document).

Table S1. Parameters of the growth strata modeling

Parameters			Interval/ increment	Total thickness/ amount of slip
Dip angle (ramp)			20°	
Steps			6	
Thickness		Growth strata	100 m	600 m
Amount of fault slip	Syncrhronous thrusting	Roof thrust	200 m	1200 m
		Ramp fault	250 m	1500 m
	Reference	Ramp fault	250 m	1500 m

Note: these parameters allow the sedimention rate to be almost equal to the uplift rate in our model, which is similar to the case study in Pishan.

Table S2. Parameters of the terrace fold modeling

Number of terraces		5
Terrace height interval		10 m
Modeling steps		4
Dip angle	Front ramp	~17°
	Rear ramp	~17°

Model setup		Amount of fault slip/m			
		Step 1	Step 2	Step 3	Step 4
Model 1	Front ramp (F1)	30	30	30	**30**
	Rear ramp (F2)	30	30	30	**30**
Model 2	Front ramp (F1)	30	30	30	**60**
	Rear ramp (F2)	30	30	30	**0**
Model 3	Front ramp (F1)	30	30	30	**0**
	Rear ramp (F2)	30	30	30	**60**
Reference	Front ramp (F1)	60	60	60	60

Note: bold text show variations in fault slip among different terrace fold models.

Table S3. Interval velocities for transforming two-way-travel time profile into depth section

Layers	Seismic velocity (m/s)
Surface	2500
11 Ma	3500
15 Ma	3800
35Ma	4000
E	4000
Carboniferous-Permian	5200
Devonian-Cambrian	5500
Proterozoic	6000

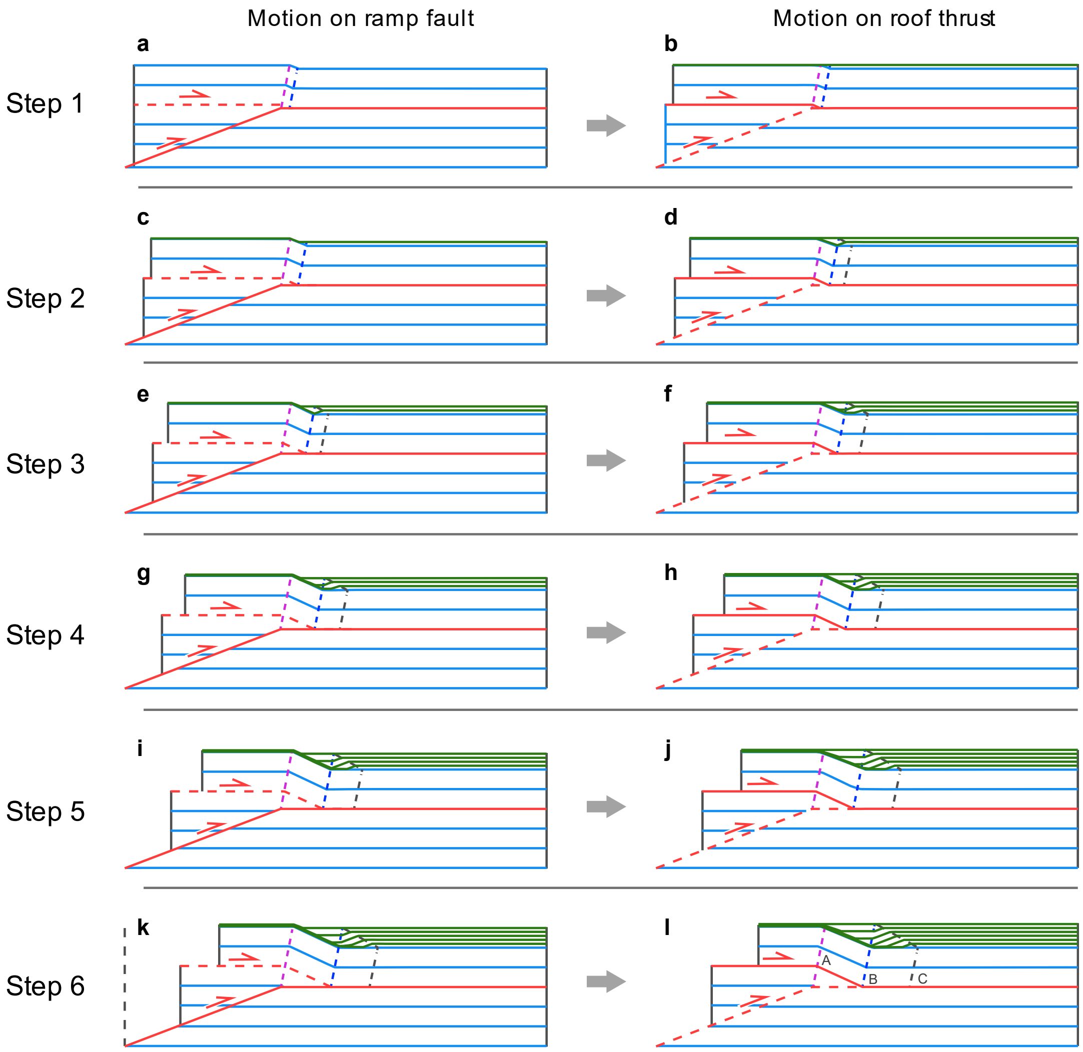

Fig. S1. Sequential modeling of growth strata during two synchronously active faults at the duplex front. We simulate the growth strata of synchronous thrusting by alternating movement between the two faults.

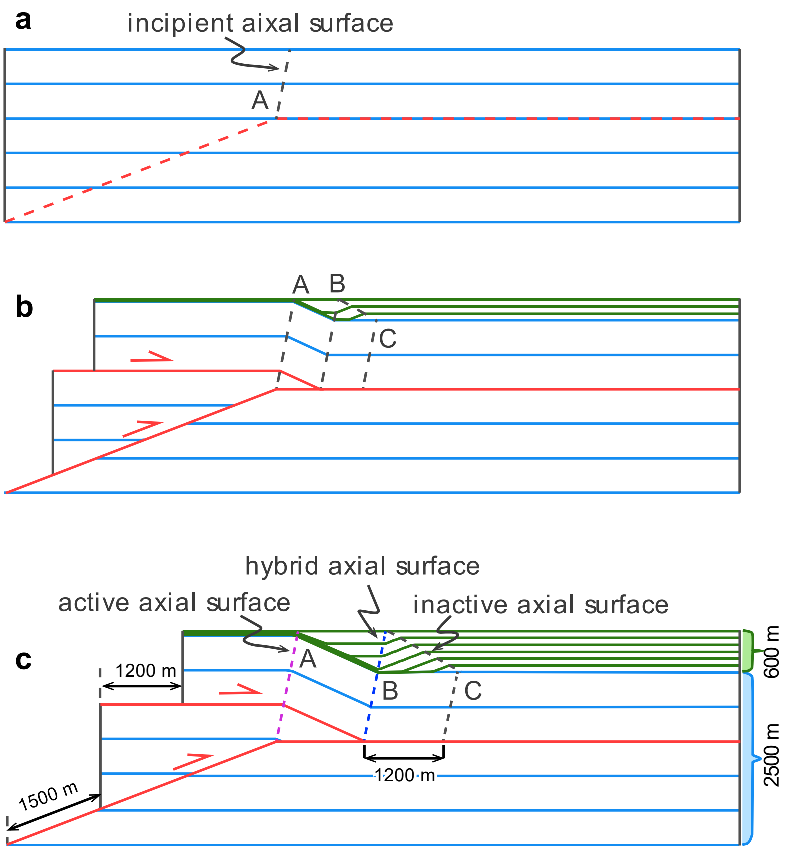

Fig. S2. Growth strata model of synchronous duplex thrusting with sharp fault bend. This figure shows parameters of the growth strata modeling.

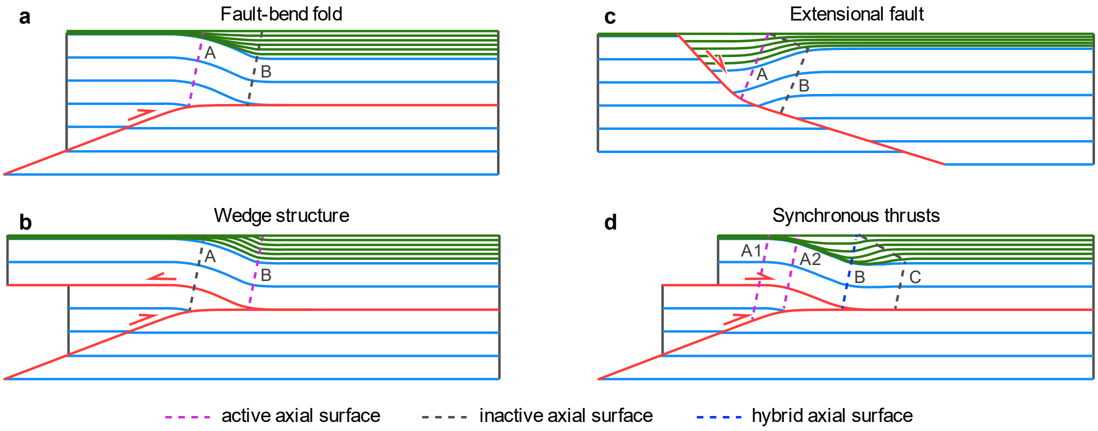

Fig. S3. Comparison of growth strata in fault-related fold with smooth fault bend. **a** Classic fault-bend fold, **b** wedge structure, **c** listric normal fault, and **d** synchronous thrusts. Note: the model of synchronous thrusting shows similar pre-growth strata geometry with the fault-bend folding and wedge structure thrusting, meanwhile it shows similar growth strata geometry with listric normal fault.

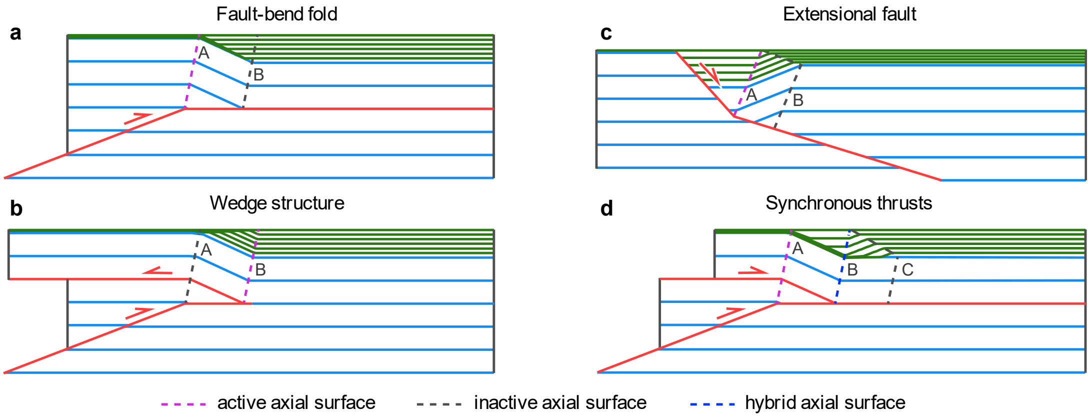

Fig. S4. Comparison of growth strata in the fault-related fold with sharp fault bend. **a** Classic fault-bend fold, **b** wedge structure, **c** listric normal fault, and **d** synchronous thrusts.

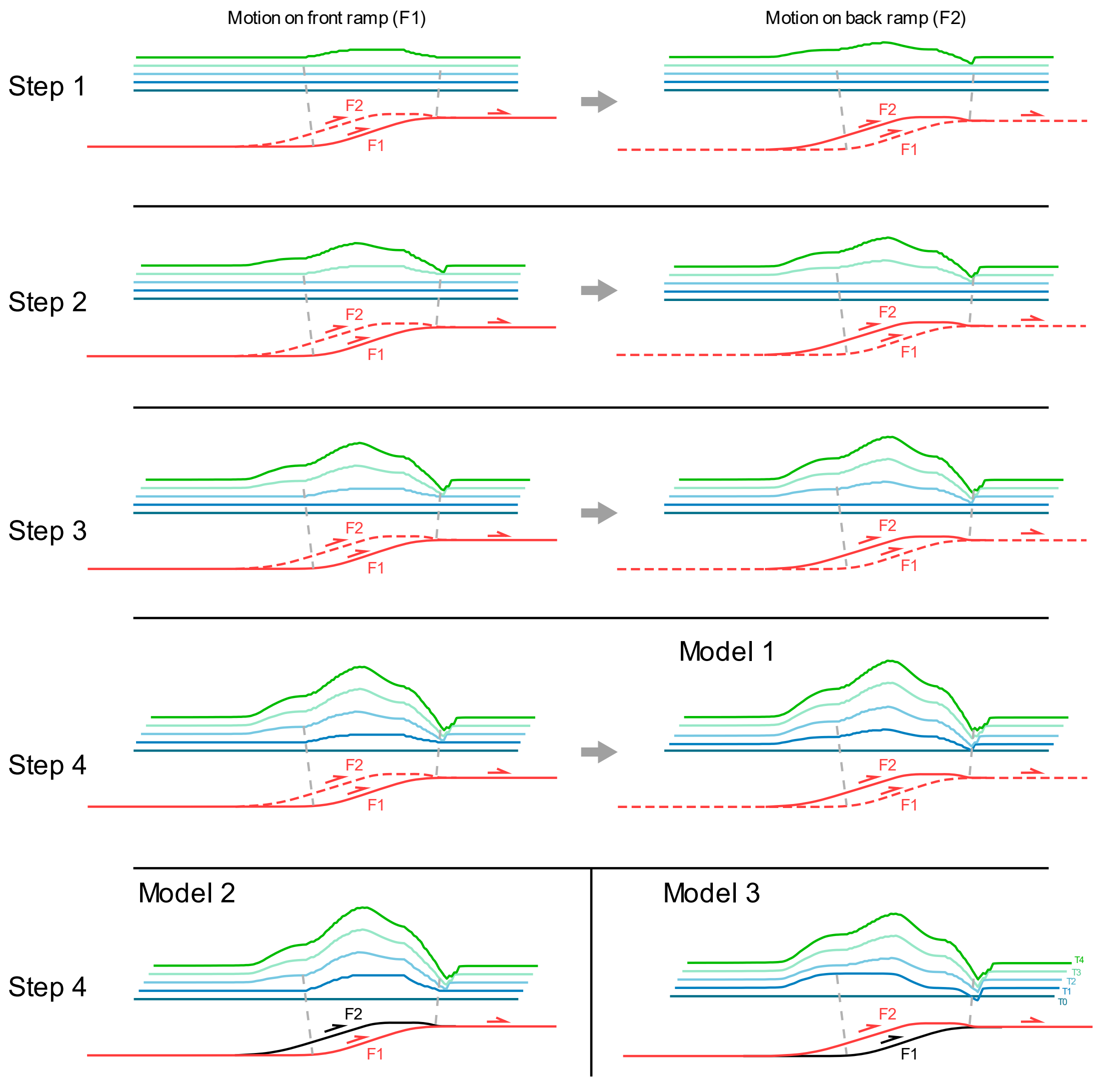

Fig. S5. Sequential modeling of terrace folds during two synchronously active faults in a duplex system. The terrace fold is shown with vertical exaggeration (×10). Model 1, Model 2 and Model 3 show differences in stage 4.

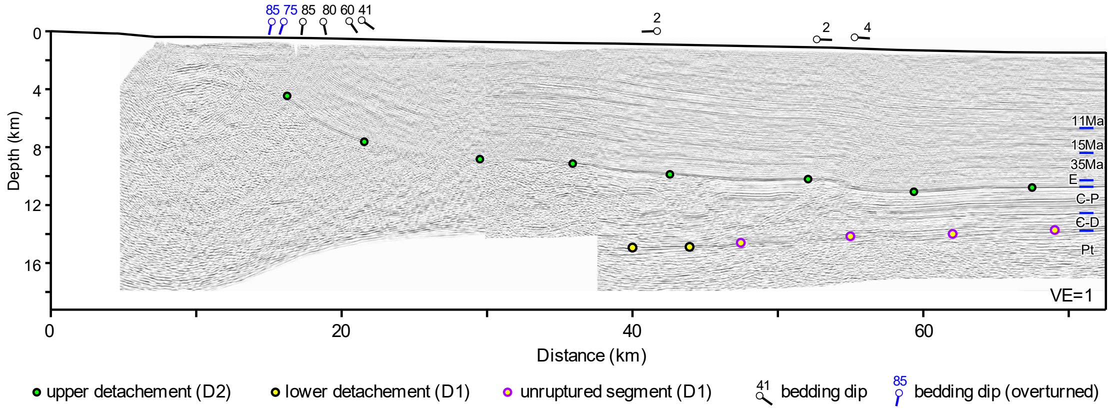

Fig. S6. Uninterpreted seismic image of the Pishan-Keliyang section. The attitude data of surface strata are from the 1:100,000 geological map of the southern Tarim Basin.

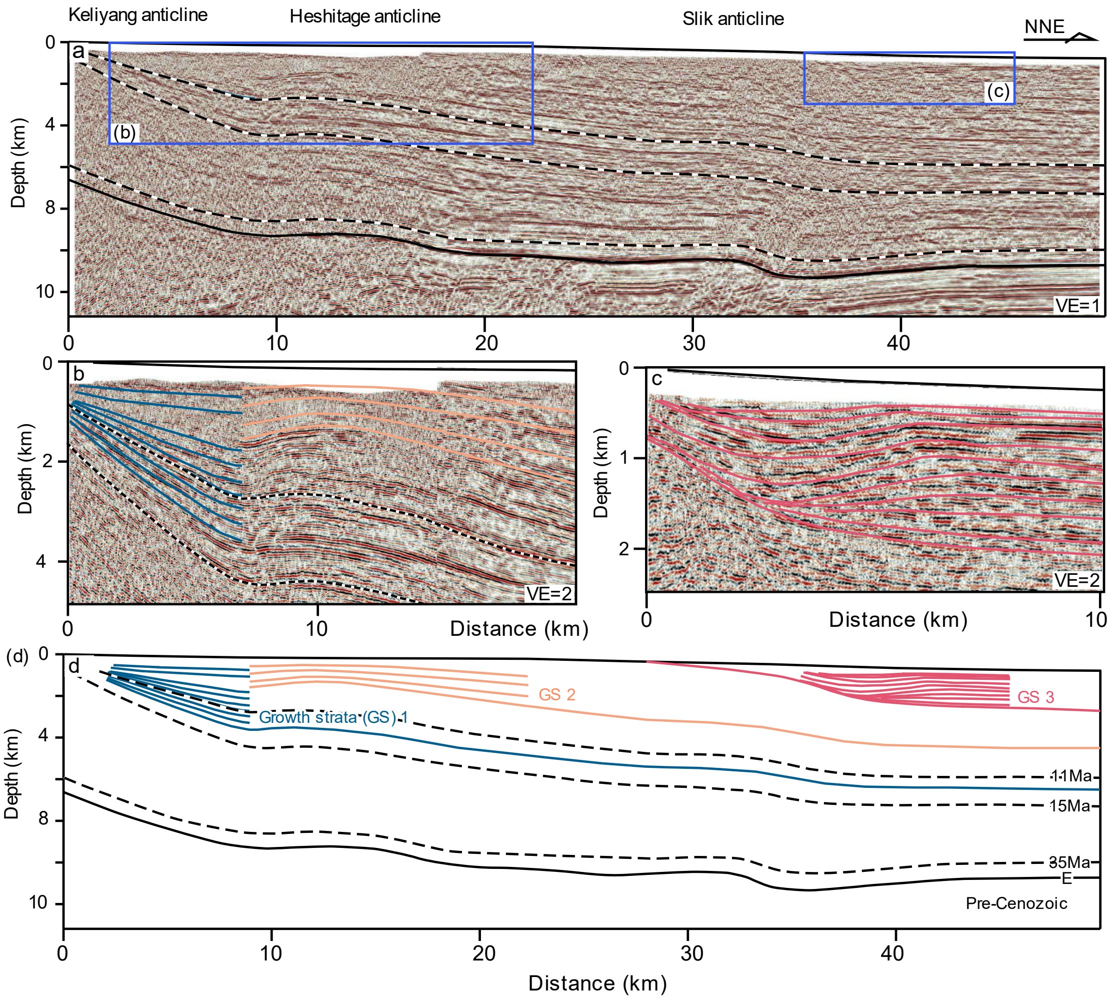

Fig. S7. Growth strata in the Pishan duplex system. a Seismic reflection profile; **b-c** enlarged seismic reflection and geologic interpretations across the Keliyang-Heshitage Anticlines (b) and Slik Monocline (c). **d** Line-drawing of seismic data shows the growth strata of the Keliyang Anticline (GS 1), Heshitage Anticline (GS 2), and Slik Monocline (GS 3). The initiation of anticlines in this profile is younging towards the basin.

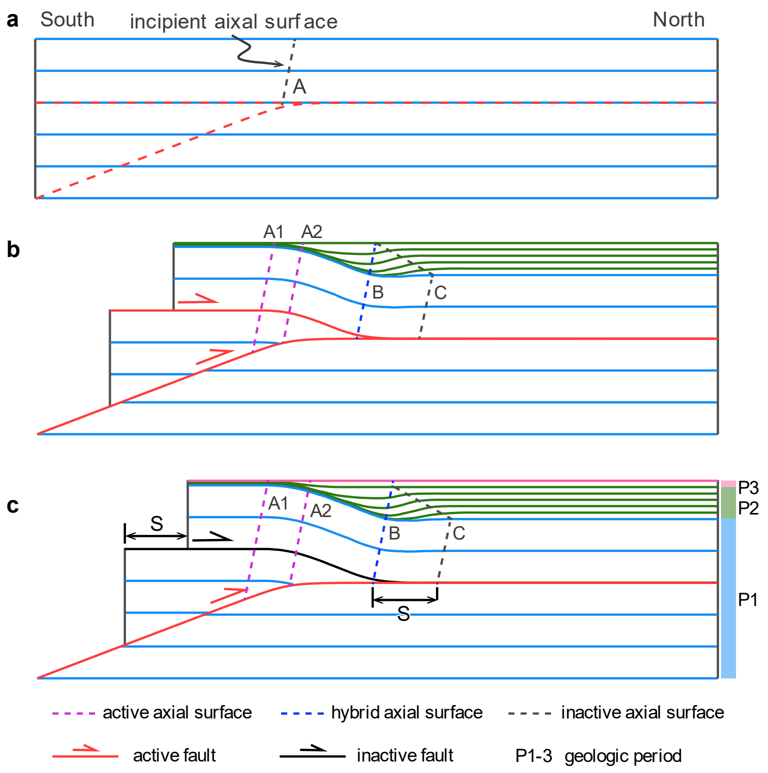

Fig. S8. Growth strata of synchronous thrusting followed by fault-bend folding. P1: quiescent period (a); P2: synchronous thrusting period (b); P3: fault-bend faulting period (c).

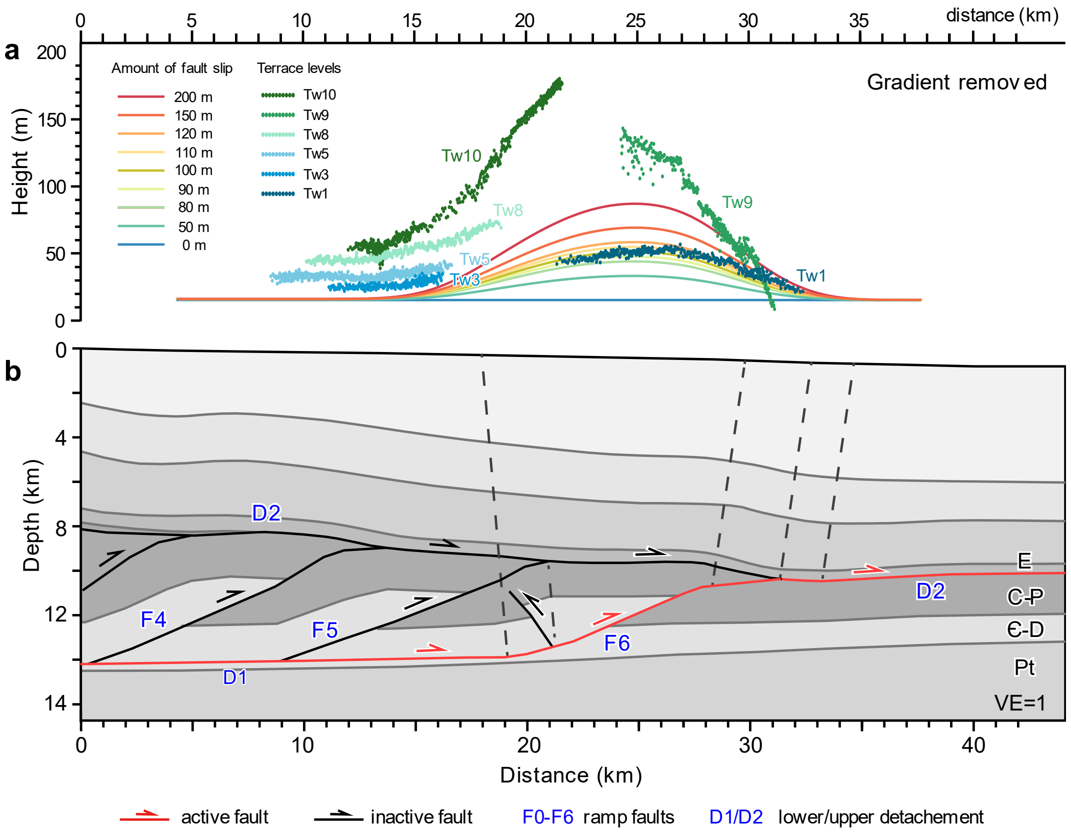

Fig. S9. Kinematic modeling of terrace deformation above the frontal ramp in the Pishan belt. a Profiles of river terraces. Data are obtained from Ainscoe et al.[2]. The curved colored lines represent the modeled terrace deformation above the frontal ramp (ref. to details in supplementary Text S3). **b** Geometry of the Pishan fold-and-thrust belt reveals the kinematic configuration with the active and inactive faults. The active frontal ramp (F6) is used to model the terrace deformation.

Supplementary References:

1. Chen H*, et al.* Using migrating growth strata to confirm a ~230-km-long detachment thrust in the southern Tarim Basin. *Journal of Structural Geology* **154**, 104488 (2022).
2. Ainscoe EA*, et al.* Blind Thrusting, Surface Folding, and the Development of Geological Structure in the Mw 6.3 2015 Pishan (China) Earthquake. *Journal of Geophysical Research: Solid Earth* **122**, 9359-9382 (2017).
