# Supplementary figures and images for: Reconciling patterns of long-term topographic growth with coseismic uplift by synchronous duplex thrusting
Source: Nat Commun. 2023 Dec 6;14:8073. doi: 10.1038/s41467-023-43994-6 (PMC10700525; doi:10.1038/s41467-023-43994-6)

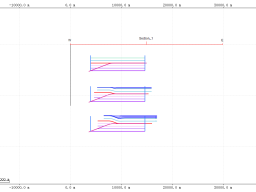

Supplement: Supplementary file 3 — Source Data [file 41467_2023_43994_MOESM3_ESM.zip › Source Data/Figure 3a.movd/thumb.png]

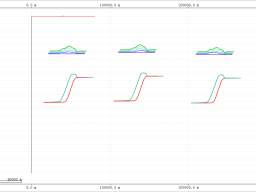

Supplement: Supplementary file 3 — Source Data [file 41467_2023_43994_MOESM3_ESM.zip › Source Data/Figure 3c-e.movd/thumb.png]

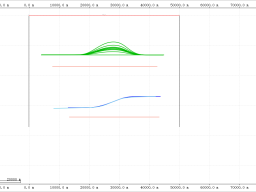

Supplement: Supplementary file 3 — Source Data [file 41467_2023_43994_MOESM3_ESM.zip › Source Data/Figure 4c and S9.movd/thumb.png]

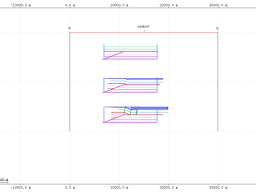

Supplement: Supplementary file 3 — Source Data [file 41467_2023_43994_MOESM3_ESM.zip › Source Data/Figure S1.movd/thumb.png]

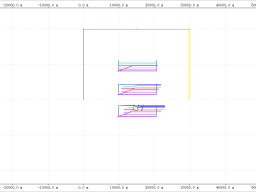

Supplement: Supplementary file 3 — Source Data [file 41467_2023_43994_MOESM3_ESM.zip › Source Data/Figure S2.movd/thumb.png]

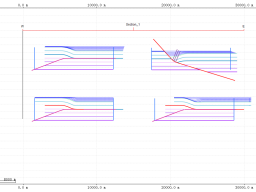

Supplement: Supplementary file 3 — Source Data [file 41467_2023_43994_MOESM3_ESM.zip › Source Data/Figure S3.movd/thumb.png]

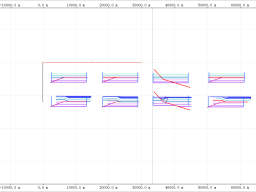

Supplement: Supplementary file 3 — Source Data [file 41467_2023_43994_MOESM3_ESM.zip › Source Data/Figure S4.movd/thumb.png]

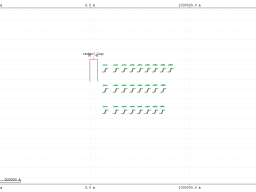

Supplement: Supplementary file 3 — Source Data [file 41467_2023_43994_MOESM3_ESM.zip › Source Data/Figure S5.movd/thumb.png]

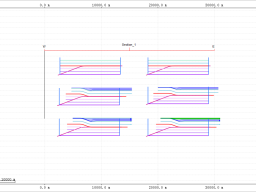

Supplement: Supplementary file 3 — Source Data [file 41467_2023_43994_MOESM3_ESM.zip › Source Data/Figure S8.movd/thumb.png]
